# Endogenous interleukin-10 constrains Th17 cells in patients with inflammatory bowel disease

**DOI:** 10.1186/1479-5876-9-217

**Published:** 2011-12-16

**Authors:** Cailin M Wilke, Lin Wang, Shuang Wei, Ilona Kryczek, Emina Huang, John Kao, Yanwei Lin, Jingyuan Fang, Weiping Zou

**Affiliations:** 1 Department of Surgery, University of Michigan, Ann Arbor, MI, USA; 2 Graduate Program in Immunology, University of Michigan, Ann Arbor, MI, USA; 3 Department of Medicine, University of Michigan, Ann Arbor, MI, USA; 4 Department of Medicine, Renji Hospital, Shanghai Jiao-Tong University, Shanghai, P. R. China; 5 University of Michigan Comprehensive Cancer Center, Ann Arbor, MI, USA; 6 Graduate Program in Cancer Biology, Ann Arbor, MI, USA

**Keywords:** Th17, IL-10, IL-1, IL-17, inflammation, Crohn's disease

## Abstract

**Background:**

Th17 cells play a role in inflammation. Interleukin (IL)-10 is a potent anti-inflammatory cytokine. However, it is poorly understood whether and how endogenous IL-10 impacts the development of Th17 cells in human pathologies.

**Materials and methods:**

We examined the relationship between IL-10 and Th17 cells in patients with Crohn's disease and in IL-10-deficient (IL-10^-/-^) mice. Th17 cells and dendritic cells (DCs) were defined by flow cytometry and evaluated by functional studies.

**Results:**

We detected elevated levels of IL-17 and Th17 cells in the intestinal mucosa of patients with Crohn's disease. Intestinal DCs from Crohn's patients produced more IL-1β than controls and were superior to blood DCs in Th17 induction through an IL-1-dependent mechanism. Furthermore, IL-17 levels were negatively associated with those of IL-10 and were positively associated those of IL-1β in intestinal mucosa. These data point toward an *in vivo *cellular and molecular link among endogenous IL-10, IL-1, and Th17 cells in patients with Crohn's disease. We further investigated this relationship in IL-10^-/- ^mice. We observed a systemic increase in Th17 cells in IL-10^-/- ^mice when compared to wild-type mice. Similar to the intestinal DCs in patients with Crohn's disease, murine IL-10^-/- ^DCs produced more IL-1β than their wild-type counterparts and promoted Th17 cell development in an IL-1-dependent manner. Finally, *in vivo *blockade of IL-1 receptor signaling reduced Th17 cell accumulation and inflammation in a mouse model of chemically-induced colitis.

**Conclusions:**

Endogenous IL-10 constrains Th17 cell development through the control of IL-1 production by DCs, and reaffirms the crucial anti-inflammatory role of IL-10 in patients with chronic inflammation.

## Introduction

Inflammation is associated with autoimmune diseases and cancer development [1,2]. Recent studies have emphasized the relevance of Th17 cell function in human diseases, including multiple sclerosis [3], colitis [4,5], psoriasis [6,7] and cancer [8,9]. It has been reported that a variety of cytokine cocktails including transforming growth factor beta (TGFβ) and the interleukins (IL)-6, IL-1, and IL-23 promote Th17 cell development [10-15], whereas IL-2 inhibits Th17 cell development [16]. It is generally accepted that these cytokines directly target T cells, where they regulate the expression of certain transcription factors and cytokine receptors, and affect Th17 cell development [17-19]. Importantly, however, effector T helper (Th) cells are polarized by antigen-presenting cells (APCs). The role of APC subsets including dendritic cells (DCs) and macrophages has not been studied in the development of Th17 cells in the microenvironment of intestinal mucosa in patients with Crohn's disease (CD). In this study, we examined the effects of Crohn's APCs and the associated cytokines on Th17 cell induction in patients with CD. We extended and confirmed our human studies in mouse model with chemically-induced intestinal inflammation.

Furthermore, we extended and confirmed our human studies in IL-10-deficient mouse model. IL-10-deficient mice show enhanced development of several inflammatory and autoimmune diseases [20], which partially micmics patients with CD. It suggests that IL-10 may serve a central function in vivo in restricting inflammatory responses in patients with CD. In support of this possibility, it was recently reported that a CD-associated NOD2 mutation suppresses transcription of human IL-10 by inhibiting activity of the nuclear ribonucleoprotein hnRNP-A1, and low IL-10 expression is associated with this mutation [21]. IL-10 is an immunosuppressive cytokine that is produced by several cell types, including myeloid APCs [22-25]. IL-10 often directly targets APCs in an autocrine manner and impedes T cell activation and polarization, thereby reducing inflammation [22,23,26-29]. Thus, it is possible that IL-10 affects the functionality of APCs, impacts Th17 cell development, and Th17-associated human pathogeneses. Thus, we assessed the role of APC-derived IL-10 in both patients with CD and IL-10^-/- ^mouse model, and investigated the cellular and molecular relationship between IL-10 and Th17 cells in these two systems. Notably, there is strong genetic evidence that IL-23 plays a role in CD. IL-23 receptor polymorphisms were strongly associated with susceptibility to CD in genome-wide scans [30]. An elevation in transcripts encoding several inflammatory cytokines including IL-6, IL-8, IL-17, IL-23 and TNFα is detected in intestinal biopsies from individuals with active CD [31]. On the basis of these results, clinical studies have begun with anti-IL-12p40 (IL-23p40) [32,33] or anti-IL-17 treatment in patients with autoimmune diseases including active CD [7]. Mixed clinical responses are reported in a variety of autoimmune diseases [7,32,33]. Our data demonstrate that endogenous IL-10, likely derived from DCs, constrains Th17 cell development through IL-1 in both scenarios. Our results and current clinical trials demonstrate that several key Th17-associated cytokines, rather than one specific cytokine (IL-17 or IL-23), play important roles in human autoimmunity. Thus, to engender reliable and efficient clinical therapeutic efficacy, small molecules, monoclonal antibodies and other recombinant receptor decoys may be designed to simultaneously target multiple crucial inflammatory mediators.

## Materials and methods

### Patients

Blood was collected from patients with Crohn's disease and healthy volunteers. Fresh colon tissues were collected from patients with Crohn's disease who underwent prophylactic colonic resections or diagnostic biopsies. Fresh "approximately normal" colon tissues adjacent to colorectal carcinoma were also collected as control tissues. All patients with Crohn's disease were in remission and were not treated with steroid drugs or antibiological therapy during the 2 months before the study. Patients involved in the study were consented, and the study was approved by local Institutional Review Boards.

### Mice

6-12-week old female and male C57BL/6 wild-type, IL-10^-/-^, and IL-1R^-/- ^mice were purchased from the Jackson Laboratory and bred in-house. This research was approved by the committee on Use and Care of Animals at the University of Michigan.

### IL-1Ra treatment

The human recombinant IL-1 receptor antagonist Anakinra was administered at 150 mg/kg to mice intraperitoneally for 8 days. Mice not receiving Anakinra were injected with PBS vehicle. For treatment of DSS-challenged mice, Anakinra administration (200 mg/kg) began on the first day of the second cycle of DSS and continued through the end of the experiment.

### DSS treatment

Mice were treated with 3% DSS in water for 5 days, followed by a rest period of 16 days during which they were allowed free access to normal water. This treatment was repeated for a total of two DSS cycles. Mice were sacrificed at the end of the second rest period, and their colons were jelly-rolled, formalin-fixed, and subjected to hematoxylin and eosin (H&E) staining.

### Flow cytometry analysis (FACS)

Single-cell suspensions were made from human and mouse tissues. Cells were labeled with fluorescence-conjugated antibodies to CD45, CD11c (both Invitrogen), CD90, CD4, CD8, IL-17, FoxP3 (all eBioscience), and/or CD3 (BD Pharmingen). For cytokine profiles, the cells were stimulated, stained and analyzed as previously published[26,34] with FacsDIVA software (BD Biosciences).

### Real-time reverse-transcriptase polymerase chain reaction (RT-PCR)

Lin^-^CD11c^+ ^cells (DCs) were isolated with a CD11c^+ ^positive selection kit (StemCell Technologies, Vancouver, British Columbia, Canada) and sorted from IL-10^+/+ ^or IL-10^-/- ^splenic cells and cultured with or without LPS stimulation. In other experiments, fresh DCs (Lin^-^CD11c^+^) or macrophages (CD14^+^) were isolated and sorted from Crohn's tissue, control colon tissue, or blood. Cytokine transcripts were detected by real-time RT-PCR as previously described [8]. Complementary DNA was normalized against and expressed as the relative values to the house keeping gene gyceraldehyde-3-phosphate dehydrogenase (*GAPDH*).

### T cell culture

Mouse spleen or human colon DCs and CD4^+ ^T cells were co-cultured in a ratio of 1:5 for 5-6 days with anti-CD3 and anti-CD28 antibodies. In other experiments, Crohn's T cells (1 × 10^6^/ml) were activated for 40 hours with anti-CD3 (5 ug/ml) and anti-CD28 (2.5 ug/ml). Colon tissue cells (2 × 10^6^/ml) from colon cancer patients were cultured for 40 hours with medium or T cell supernatants in the presence of anti-IL-17R antibody (R&D, clone 133617) or isotype control. Cytokines in cell culture supernatants were detected by ELISA.

### ELISA cytokine detection

Supernatant was collected from culture with mouse T cells and DCs, or LPS-activated DCs, or human colon lamina propria or CD4^+ ^T cells, or human colon cells and Crohn's T cells co-culture. In other experiments, fresh serum was collected from the blood of healthy volunteers or Crohn's disease patients. Cytokines were detected using murine or human DuoSet kits (R&D Systems, Minneapolis, MN).

### Statistics

Experiments were evaluated using the Mann-Whitney or Chi-squared test, with P < 0.05 considered significant. Statistics were performed in the GraphPad Prism program suite (GraphPad Software, Inc., La Jolla, CA) and the Statistica program suite (StatSoft, Tulsa, OK).

## Results

### Increased IL-17 and Th17 cells in the intestine of patients with Crohn's disease

**Figure 1:**
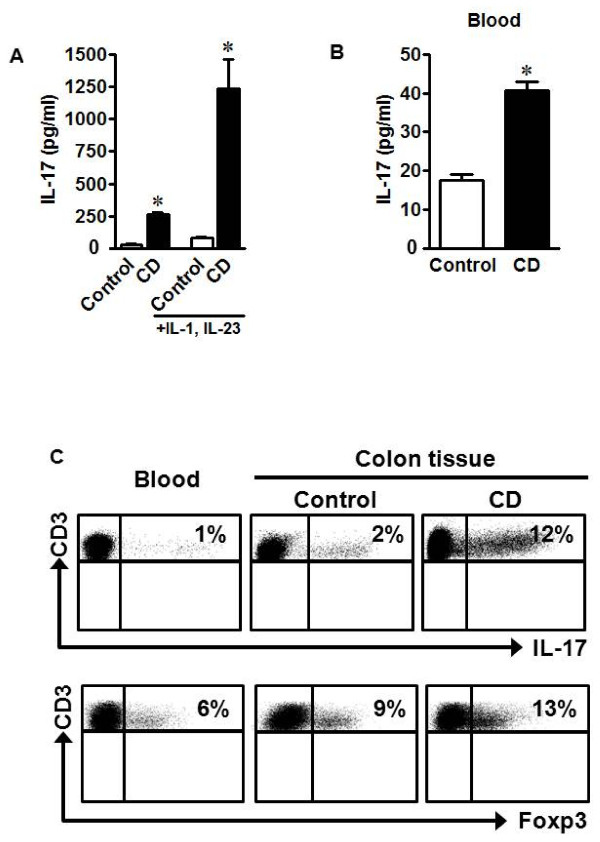
**Th17 cells and IL-17 are increased in CD patient tissue**. a) Lamina propria mononuclear cells from control colon tissues or CD colons were cultured for two days with IL-1 and IL-23. Supernatants were subjected to ELISA for analysis of IL-17 protein. b) Serum from healthy or CD patients was subjected to ELISA for IL-17 quantification. n = 16. *P < 0.0001. c) Healthy blood or control colon tissue or CD colon tissue cells were stained with antibodies to CD3, CD4, IL-17, and FoxP3 and antigen expression was analyzed via FACS. Results are expressed as the percent of IL-17^+ ^or Foxp3^+ ^T cells in the CD4^+ ^T cells. The gates were on CD4^+^CD3^+ ^T cells for both Th17 and Treg cells. n = 16.

To investigate if intestinal T cells in patients with Crohn's disease (CD) included Th17 cells, we isolated the lamina propria mononuclear cell fraction from fresh CD colonic tissues or from "approximately normal" adjacent colonic tissues in patients with colorectal cancer. We detected higher levels of IL-17 in the supernatant of briefly cultured CD lamina propria mononuclear cells than in the supernatant from control lamina propria cells (Figure 1a). This indicates that IL-17 was spontaneously released in the cultures of CD-associated immune cells. After a short stimulation with IL-1 and IL-23, the levels of IL-17 were further increased in cultures of lamina propria mononuclear cells from CD patients and from control patients. However, the levels of IL-17 were 15 times higher in patients with CD than control (Figure 1a). Interestingly, limited levels of IL-17 were detected in peripheral blood from healthy controls, but were substantially increased in CD patients (Figure 1b). Intracellular staining for IL-17 revealed that IL-17 expression was in CD4^+^CD3^+ ^cells. In line with the data on IL-17 in culture supernatants (Figure 1a, b), the numbers of Th17 cells and Foxp3^+^CD4^+ ^regulatory T cells were significantly higher in the CD colon tissues than in peripheral blood and the colon tissue from non-CD patients (Figure 1c). The data suggest that Th17 cells may be induced in the local pathological environment in CD patients.

### Th17 cells are associated with reduced IL-10 and increased IL-1 in CD patients

**Figure 2:**
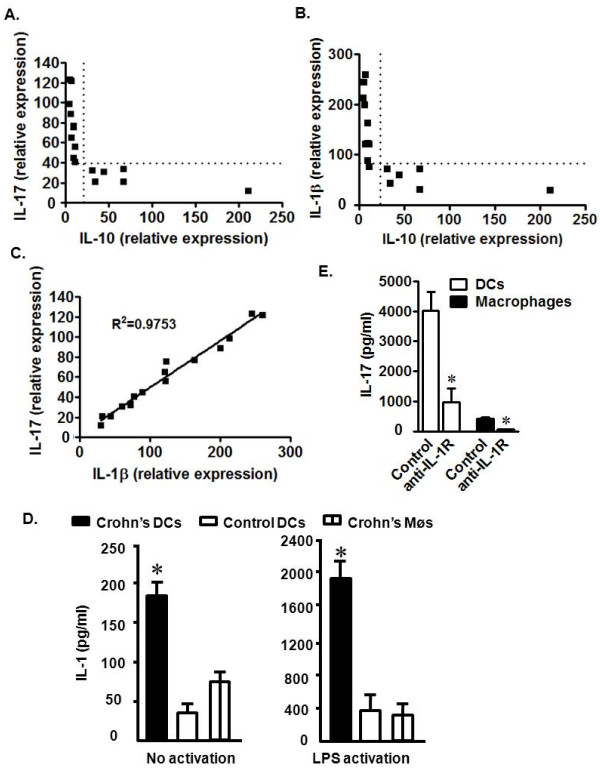
**Relationships among IL-17, IL-1, and IL-10 in CD patients**. a-c) IL-17, IL-10, and IL-1β message from fresh CD mononuclear lamina propria cells were quantified via real-time PCR. Chi-squared (χ^2^) test. P < 0.0001 for the following comparisons: IL-17 vs IL-10 (a), IL-1β vs IL-10 (b), and IL-17 vs IL-1β (c). n = 16. d) Crohn's DCs expressed high levels of IL-1β. Crohn's DCs and macrophages, and normal colon DCs were cultured for 40 hours with or without LPS. Normal control DCs were isolated from normal colon tissues at least 5 cm away from colon cancer. IL-1β protein was quantified via ELISA. n = 6. *, P < 0.005. e) Crohn's DCs induced high levels of T cell-derived IL-17 production through IL-1. CD4^+ ^T cells were cultured for 48 hours with Crohn's myeloid DCs or macrophages in the presence anti-IL-1R. IL-17 protein was detected via ELISA in supernatants. n = 6. *, P < 0.005.

We next examined the potential mechanisms by which Th17 cells were increased in the local CD environment. IL-10 gene polymorphisms that result in defective IL-10 production are observed in patients with CD [21]. We quantified IL-17 and IL-10 transcripts in mononuclear lamina propria cells from patients with CD. The level of IL-10 messages were negatively associated with those of IL-17 (Figure 2a). We hypothesized that decreased IL-10 in CD patients may allow increased local concentrations of cytokines associated with Th17 cell development. Accordingly, we further quantified TGFβ, IL-1α, IL-1β, IL-6, and IL-23 in mononuclear lamina propria cells from patients with CD. The levels of TGFβ, IL-1α, IL-6, and IL-23 were not associated with that of IL-10 (not shown). To our surprise, we observed a significant negative association between IL-1β and IL-10 (Figure 2b). Not surprisingly, however, was the observation that IL-1β and IL-17 message levels correlated directly (Figure 2c). Given the importance of myeloid APC-derived IL-1 in the induction of human Th17 cells[8], we theorized that myeloid APC subsets induced Th17 cells through IL-1 in CD tissues. To test this hypothesis, we demonstrated that both CD macrophages and myeloid dendritic cells (DCs) spontaneously released IL-1β, and stimulation with lipopolysaccharide (LPS) further increased the levels of IL-1β (Figure 2d). However, regardless of stimulation, Crohn's intestinal DCs produced higher levels of IL-1β than Crohn's intestinal macrophages and normal intestinal DCs (Figure 2d). This indicates that DCs from CD patients have an increased potential to release IL-1β into the intestinal milieu. Consistent with this, we observed that CD DCs induced higher levels of T cell IL-17 production than macrophages. Blockade of IL-1 receptor signaling reduced CD4^+ ^T cell-derived IL-17 production induced by both DCs and macrophages (Figure 2e). The data suggest that reduced IL-10 expression by intestinal DCs may promote Th17 cell development through increased IL-1 production in patients with CD.

### Th17 cells promote inflammation in CD patients

**Figure 3:**
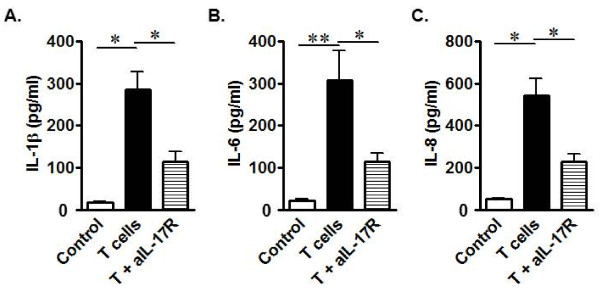
**Crohn's disease T cell-derived IL-17 stimulates the production of inflammatory cytokines**. "Normal" colon tissue cells were cultured for 40 hours with medium alone or CD T cell supernatants with or without anti-IL-17R. IL-1β (a), IL-6 (b), and IL-8 (c) were quantified in supernatant. n = 5. *, P < 0.05 and **, P < 0.01.

Th17 cells may play a role in the pathogenesis of CD [4,35-37]. To directly demonstrate the inflammatory functionality of intestinal Th17 cells, we sorted and activated intestinal CD4^+ ^T cells from patients with Crohn's disease. Fresh colon tissue cells were exposed to supernatants from activated CD T cells for a short time. In the absence of CD T cell supernatant, colon tissue cells produced minimal amounts of IL-1β (Figure 3a), IL-6 (Figure 3b), and IL-8 (Figure 3c). Interestingly, the levels of IL-1β, IL-6, and IL-8 protein were dramatically increased in the presence of CD T cell supernatants (Figure 3a-c). Blockade of IL-17 receptor (IL-17R) reduced the production of IL-1β, IL-6, and IL-8 stimulated by CD T cell supernatant (Figure 3a-c), suggesting that IL-17 is at least partially responsible for the production of these inflammatory cytokines. Human CD Th17 cells are therefore functional and may play an active pro-inflammatory role in the local disease environment. Thus far, the *ex vivo *and *in vitro *data support a cellular and molecular link among IL-10, IL-1β, and Th17 cells in humans with CD.

### IL-10^-/- ^murine DCs are superior Th17 cell inducers

**Figure 4:**
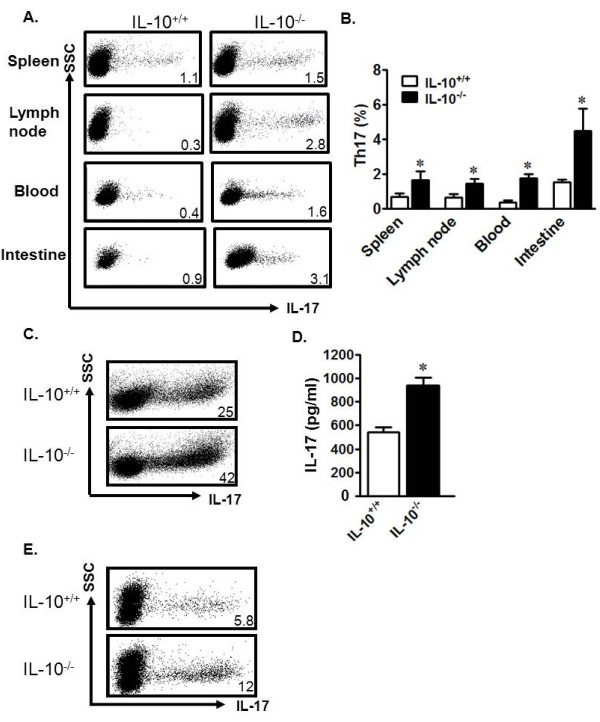
**Murine IL-10^-/- ^DCs are powerful Th17 inducers**. a, b) Increased Th17 cells in IL-10^-/- ^mice. Mononuclear cells from unchallenged IL-10^+/+ ^and IL-10^-/- ^mouse organs were analyzed via FACS. a) Representative FACS plots. b) Results are expressed as the percentage of Th17 cells in CD4^+ ^T cell populations ± SEM. 6 mice/group, *P < 0.05. c, d). IL-10^-/- ^spleen T cells were susceptible to Th17 polarization. IL-10^+/+ ^and IL-10^-/- ^spleen T cells were polarized with Th17-polarizing cytokines for 6 days. Th17 cells were analyzed by FACS. c) Results are expressed as the percentage of Th17 cells in CD4^+ ^T cells. d) Supernatant from Th17 cultures was collected on day 3 and analyzed via ELISA for levels of IL-17. *P < 0.05, average of three experiments. e) IL-10^-/- ^DCs were potent Th17 inducers. IL-10^+/+ ^CD4^+ ^T cells were cultured with IL-10^-/- ^or IL-10^+/+ ^splenic DCs in a ratio of 5:1 for 5 days. Th17 cells were analyzed by FACS. Results are expressed as the percentage of Th17 cells in CD4^+ ^T cells. n = 6, *P < 0.05. Y axis showed SSC and the dot plots were gated on CD4^+^CD3^+ ^T cells (a, c, e).

IL-10^-/- ^mice develop chronic enterocolitis, a condition that shares many architectural signatures with human CD [20]. These similarities include increased numbers of focal ulcerations and transmural lesions, as seen in CD patients [38]. To overcome the experimental limitations of mechanism investigation using limited tissue from CD patients, we extended our studies to the IL-10^-/- ^mouse model. We observed increased levels of Th17 cells in the lymph nodes, spleen, blood, and intestines of IL-10^-/- ^mice as compared to wild-type (IL-10^+/+^) mice (Figure 4a, b). There were no differences in other immune cell subsets, including B cells, macrophages, DCs, granulocytes, and natural killer cells between IL-10^-/- ^and IL-10^+/+ ^mice (not shown). Thus, Th17 cells are spontaneously and systemically increased in IL-10^-/- ^mice.

We next studied the potential underlying mechanisms causing the spontaneous increase of Th17 cells in IL-10^-/- ^mice. To this end, IL-10^+/+ ^and IL-10^-/- ^spleen T cells were cultured under Th17-polarizing conditions. We observed that there were more Th17 cells in the IL-10^-/- ^cultures (Figure 4c) and more IL-17 in the IL-10^-/- ^culture supernatant when compared to IL-10^+/+ ^cultures (Figure 4d). To examine the cellular cause of the increased Th17 cells, we investigated the role of IL-10^-/- ^DCs in Th17 cell induction. We co-cultured wild-type T cells with wild-type DCs or IL-10^-/- ^DCs, and examined the resulting cellular phenotypes. We found increased Th17 cells in co-cultures with IL-10^-/- ^DCs as compared to those with wild-type DCs (Figure 4e). Thus, similar to human Crohn's DCs, IL-10^-/- ^mouse DCs are superior inducers of Th17 cells.

### IL-10^-/- ^DCs induce Th17 cells through IL-1 in mice

**Figure 5:**
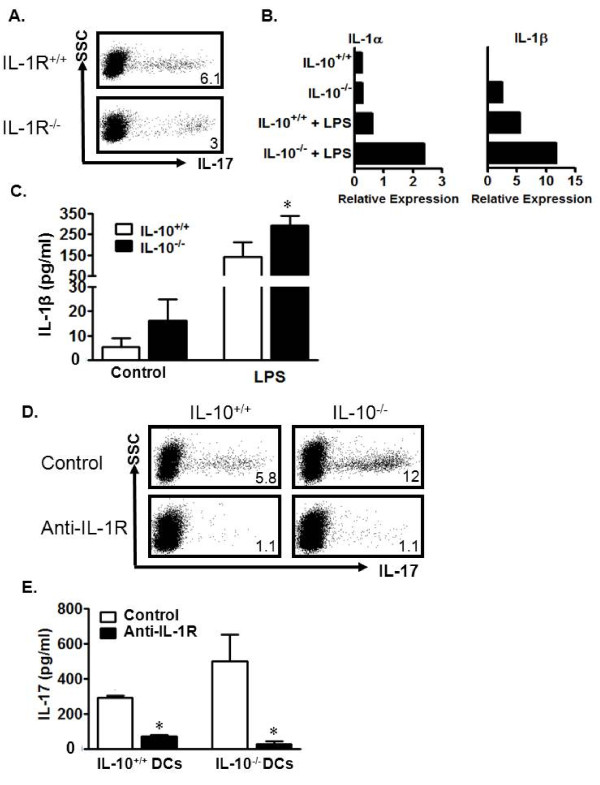
**Mouse IL-10^-/- ^DCs induce Th17 cells through the IL-1/IL-1R signaling pathway**. a) Reduced Th17 cells in DC-stimulated IL-1R^-/- ^CD4^+ ^T cell cultures. IL-1R^-/- ^or IL-1R^+/+ ^CD4^+ ^T cells were stimulated with wild-type DCs for 5 days. The cells were analyzed for Th17. n = 3. b, c)IL-10^-/- ^DCs expressed high levels of IL-1. IL-10^+/+ ^or IL-10^-/- ^DCs were cultured with or without LPS for 8 (b) or 48 hours (c). IL-1α and IL-1β message was quantified via real-time PCR (b). IL-1β protein was detected in culture supernatant via ELISA (c). *P < 0.05, average of 3 experiments. d, e) Blockade of IL-1R reduced DC-mediated Th17 induction. IL-10^-/- ^or IL-10^+/+ ^DCs were cultured in a ratio of 1:5 with IL-10^+/+ ^CD4^+ ^T cells with or without anti-IL-1R. The cells were analyzed on day 5 for Th17. Results are expressed as the percentage of Th17 cells in CD4^+ ^T cells (d). IL-17 was detected in culture supernatants on day 3 via ELISA. Results are expressed as the mean values ± SEM (e). n = 4. *, P < 0.05. Y axis showed SSC and the dot plots were gated on CD4^+^CD3^+ ^T cells (a, d).

We next examined possible reasons why murine IL-10^-/- ^DCs are better at Th17 cell induction. We and others have demonstrated the importance of IL-1 in the development of mouse and human Th17 cells[16,39]. Human Crohn's DCs produced more IL-1, and potently induced Th17 cells (Figure 2d, e). We thus hypothesized that mouse IL-10^-/- ^DCs produce more IL-1 which in turn leads to more potent Th17 induction. We first tested this hypothesis in a co-culture system where wild-type DCs were incubated with either wild-type IL-1 receptor (IL-1R^+/+^) or IL-1R^-/- ^T cells. The resultant IL-1R^-/- ^T cell population expressing IL-17 was only half the size of that in the IL-1R^+/+ ^T cell cultures (Figure 5a). This observation confirms the importance of IL-1 signaling in Th17 development. In line with this finding, we observed increased expression of IL-1α, IL-1β, IL-6, and TNFα transcripts in IL-10^-/- ^DCs when compared to wild-type DCs (Figure 5b and not shown). Increased IL-1β protein was also detected in LPS-stimulated IL-10^-/- ^DC culture supernatants (Figure 5c).

In order to determine whether this increased IL-1 was involved in the stronger Th17 induction documented in our experiments with IL-10^-/- ^DCs, we added anti-IL-1R to the co-cultures of T cells with IL-10^+/+ ^or IL-10^-/- ^DCs. Blockade of IL-1R resulted in significantly decreased Th17 cells in both cultures (Figure 5d) and decreased IL-17 levels in culture supernatants (Figure 5e). These data indicate that IL-10^-/- ^DCs may release high levels of IL-1 and efficiently promote Th17 cell development.

### In vivo blockade of IL-1R signaling decreases Th17 cells and reduces inflammation

**Figure 6:**
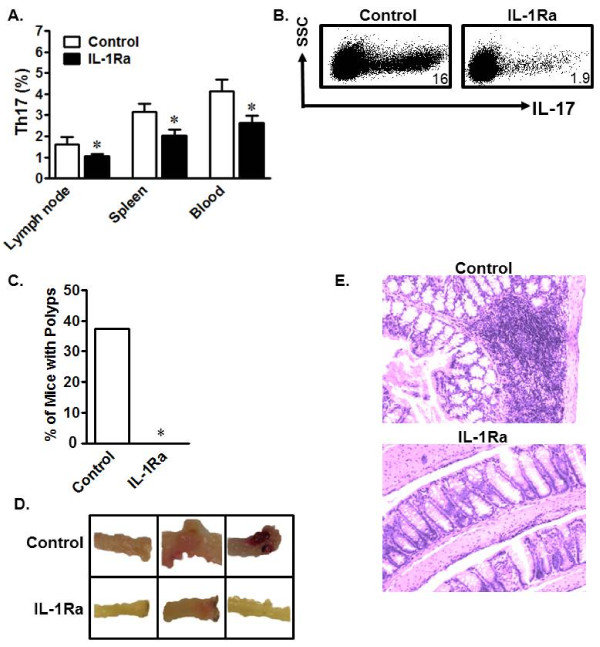
**In vivo IL-1R blockade reduces Th17 cells and inflammation**. a) In vivo IL-1R blockade decreased homeostatic Th17 cells. Mice were treated with Anakinra or PBS for 7 days. Single-cell suspensions from different organs were analyzed for Th17 cells. Results are expressed as the percentage of Th17 cells in CD4^+ ^T cells ± SEM. *P < 0.05, 5 mice/group. b) In vivo IL-1R blockade reduced DSS-induced Th17 cells. Mice were treated with DSS to induce inflammation and given Anakinra throughout the second cycle of treatment. Single-cell suspensions from different organs were analyzed for Th17 cells. Results are shown as the percentage of Th17 cells in mensenteric lymph node CD4^+ ^T cells in mice treated with Anakinra or PBS. Y axis showed SSC and the dot plots were gated on CD4^+^CD3^+ ^T cells. n = 4. c-e) In vivo IL-1R blockade prevented polyp formation and ameliorated inflammation induced by DSS. Colon polyps were counted in DSS-challenged IL-10^-/- ^mice treated with PBS or Anakinra (IL-1Ra). n = 8 mice per group, *P < 0.05 (c). Bloody and polyp-carrying colon sections from untreated mice (top) are shown, but were not observed in IL-1Ra-treated mice (bottom) (d). H&E staining showed heavy cellular infiltration in colon tissue from untreated mice (top), but not in colon tissue from IL-1Ra-treated mice (bottom) (e).

Finally, we investigated whether *in vivo *blockade of IL-1 signaling had any impact on Th17 cell development. Initially, we administered the human recombinant IL-1R antagonist (IL-1Ra) Anakinra, already shown to have efficacy in mice [40], to IL-10^+/+ ^and IL-10^-/- ^mice and analyzed Th17 cells in different organs. As expected, *in vivo *IL-1 blockade had no significant effect on Th17 cells in wild-type mice (not shown), but decreased Th17 cells in multiple compartments including lymph nodes, spleen, and peripheral blood in IL-10^-/- ^mice (Figure 6a). IL-10^-/- ^C57BL/6 mice usually do not develop colitis in pathogen-free conditions. To evaluate the link among IL-1, IL-10, and Th17 cells in an active inflammatory environment *in vivo*, IL-1Ra was administered during the course of dextran sodium sulfate (DSS)-induced colitis in IL-10^-/- ^mice. Similarly, we observed that IL-1Ra treatment decreased Th17 cells in multiple organs, including the mesenteric lymph nodes (mLN) (Figure 6b). More interestingly, Anakinra-treated mice did not develop colon polyps, while untreated mice did (Figure 6c). Grossly-evaluated colons from untreated mice exhibited more inflammation and blood vessel involvement than Anakinra-treated mice (Figure 6d). Hematoxylin and eosin (H&E)-stained sections of mouse colons showed that Anakinra-treated mice had lower inflammatory infiltrate than untreated mice (Figure 6e). These data demonstrate that IL-1 signaling blockade reduces Th17 cells and ameliorates chemically-induced inflammation. Our work therefore demonstrates a potent cellular and molecular link between IL-10 and Th17 cells in mice and in humans with Crohn's disease.

## Discussion

In the present study, we established a cellular and molecular relationship among IL-1, IL-10, and Th17 cell development in inflammatory disease models in humans and mice. This link may be important in the regulation of immune pathogenesis of human chronic inflammatory conditions, including CD.

Th17 cells play a role in the inflammatory response associated with multiple human autoimmune diseases [4,41-43] and cancer [8,9]. Th17 cells and/or IL-17 are detected in CD patients [35-37,44-48]. However, the generation and functional relevance of Th17 cells remains poorly understood in CD patients. We have tested the functionality of fresh CD Th17 cells, and found that these T cells induce the production of IL-1, IL-6, and IL-8 by colon tissue cells through IL-17 in vitro. It suggests that these cells may mediate or/and accelerate local inflammation by inducing inflammatory cytokine production. In line with this, elevated inflammatory cytokines are detected in the freshly isolated colon environment from patients with CD. It has been reported that recombinant IL-17 induces IL-6 expression in other systems [49-52]. As IL-1, IL-6, and IL-8 play crucial roles in CD [53-57], it is likely that Th17 cells promote the production of inflammatory cytokines and contribute to the immunopathogenesis of CD in patients. Notably, although IL-17, as a signature gene for Th17 cells, importantly attributes to Th17 cell biology, it is well appreciated that Th17 cell biology may depend on the synergistic effects between Th17-associated cytokines, rather than IL-17 alone [8,58]. For example, IL-17 and IFNγ synergistically induce β-defensin expression in patients with psoriasis [6] and Th1-type chemokine production in patients with cancer [8]. This may partially explain why IL-17 signaling blockade generates variable clinical benefits in patients with psoriasis, rheumatoid arthritis, and uveitis [7] and minimal clinical response in patients with CD.

We have demonstrated a cellular and molecular link among IL-10, IL-1, and Th17 cells in patients with CD and in IL-10^-/- ^mice. In CD patients, the levels of intestinal IL-10 are negatively associated with IL-17 and IL-1. Both IL-10^-/- ^mouse DCs and human Crohn's DCs are superior inducers of Th17 cells via their increased IL-1 production. Blockade of the IL-1 signaling pathway reduces Th17 cell development both *in vitro *and *in vivo*. In agreement with these observations, we and others have shown that IL-1 is crucial for inducing Th17 cells in humans and mice [16,59]. In patients with psoriasis, psoriatic DCs potently induce Th17 cells in an IL-1-dependent manner [6]. Human tumor-associated macrophages also promote Th17 cell development through IL-1 [8]. IL-1 has been shown to induce gastric inflammation and is associated gastric carcinoma [1]. Notably, IL-10 suppresses IL-1 production [60,61] and that IL-1 is involved in controlling Th17 cells in the mouse model of experimental autoimmune encephalomyelitis (EAE) [39]. Exogenous IL-10 can suppress the *in vitro *development of Th17 cells from CD4^+ ^T cells in patients with rheumatoid arthritis [62]. However, our study is the first to demonstrate a role for IL-10 in Th17 development through the control of IL-1 expression by DCs in both mouse and human systems, including CD patients. In support of our studies in patients with CD, one recent report demonstrates that mouse Th17 cells expressed interleukin-10 receptor α (IL-10Rα) *in vivo*. Importantly, T cell specific blockade of IL-10 signaling led to a selective increase of Th17 cells during intestinal inflammation in the small intestine in mice. Furthermore, in this mouse model, Treg cells were able to control Th17 cells in an IL-10-dependent manner *in vivo*. Thus IL-10 signaling directly suppresses Th17 cells [63]. However, high levels of Treg cell infiltration are detected in patients with CD and ulcerative colitis [64]. Although Treg cells inhibit Th17 cells in patients with cancer [8], it appears that human Treg cells failed doing so in the microenvironments of chronic graft-versus-host disease (GVHD), ulcerative colitis, and inflammation-associated colon cancer [9,64]. It is possible that human Th17 cells have stem cell features and are resistant to apoptosis in the chronic inflammatory microenvironments [9,64]. Nonetheless, our data indicate that IL-1 plays a key role in Th17 cell development in human autoimmune disease, and support the notion that IL-1 signaling blockade is a potential strategy to treat patients with these conditions. IL-10, via its downregulation of IL-1, is thus able to limit development of Th17 cells in mice and humans, and in doing so executes some of its anti-inflammatory effects.

The next logical step is to investigate how IL-10 controls IL-1 production by APCs. IL-10 dampens MyD88-dependent signaling in DCs and leads to LPS hyporesponsiveness [65]. Because IL-1 signaling can be mediated by MyD88, this may explain how IL-10 controls endotoxin-induced IL-1 production. It is also possible that IL-10 controls IL-1 expression machinery, such as IL-1 converting enzyme (ICE) and components of the inflammasome [66,67]. However, it remains to be determined if IL-10 suppresses IL-1 production induced by other stimuli, including the necrotic tissue often found in a chronic inflammatory environment. The key question remaining is why IL-10 production is reduced in some CD patients. A nucleotide-binding oligomerization domain containing 2 (NOD2) mutation commonly observed in CD patients may lead to inhibition of IL-10 transcription [21]. However, we have not examined the gene profile of NOD expression in our patient populations. Since 30% of CD patients have NOD mutations, it is likely that alterations in NOD2 transcription may at least partially contribute to the reduced IL-10 production in our patient tissues. The data further suggests that IL-10 therapy or IL-1 signaling blockade may not be generally meaningful for all the CD patients.

In summary, we have demonstrated that IL-10 targets APCs, and suppresses Th17 cell development in mice and humans through modulation of IL-1 production. The data document a cellular and molecular link among IL-10, IL-1, and Th17 cells, and suggest that IL-10 may inhibit inflammation via control of Th17 cell development.

## Conclusions

To date, it is well accepted that Th17 cells play a role in inflammation including Crohn's disease. Our studies have demonstrated that IL-1 drives the immune effector status towards IL-17, and dendritic cell-derived IL-10 constraints Th17 cell development through IL-1 in patients with CD. This is the first report demonstrating cellular and molecular mechanistic link among IL-1, IL-10 and Th17 cells in patients with CD. This link is functionally examined in mouse model. Demonstration of the novel mechanistic interplay between inflammatory and anti-inflammatory elements increases our understanding of CD pathogenesis and can identify novel pathways involved in disease aetiology.

## Abbreviations

Th: T-helper; Treg: regulatory T; IL: interleukin; APC: antigen-presenting cell; DC: dendritic cell; Crohn's disease: CD.

## Competing interests

The authors declare that they have no competing interests.

## Authors' contributions

CMW, LW, IK and SW performed experiments. EH, JK and WZ obtained funding, provided material and intellectual support. CMW and WZ wrote the paper. All authors read and approved the final manuscript. CMW, LW and SW contributed equally to this work.
